# Exposure to Radiofrequency Electromagnetic Field in the High-Frequency Band and Cognitive Function in Children and Adolescents: A Literature Review

**DOI:** 10.3390/ijerph17249179

**Published:** 2020-12-08

**Authors:** Toru Ishihara, Keiko Yamazaki, Atsuko Araki, Yuri Teraoka, Naomi Tamura, Takashi Hikage, Manabu Omiya, Masahiro Mizuta, Reiko Kishi

**Affiliations:** 1 Center for Environmental and Health Sciences, Hokkaido University, Sapporo 060-0812, Japan; tishihara@people.kobe-u.ac.jp (T.I.); kyamazaki@cehs.hokudai.ac.jp (K.Y.); AAraki@cehs.hokudai.ac.jp (A.A.); yteraoka@cehs.hokudai.ac.jp (Y.T.); ntamura@cehs.hokudai.ac.jp (N.T.); 2 Graduate School of Human Development and Environment, Kobe University, Kobe 657-8501, Japan; 3 Faculty of Information Science and Technology, Hokkaido University, Sapporo 060-0814, Japan; hikage@wtemc.ist.hokudai.ac.jp; 4 Information Initiative Center, Hokkaido University, Sapporo 060-0811, Japan; omiya@iic.hokudai.ac.jp (M.O.); mizuta@iic.hokudai.ac.jp (M.M.)

**Keywords:** cognition, intelligence, memory, mobile phone, RF-EMF

## Abstract

With increasing use of mobile phones, exposure to radiofrequency electromagnetic field (RF-EMF) in the high-frequency band associated with mobile phones has become a public concern, with potentially adverse effects on cognitive function in children and adolescents. However, findings regarding the relation of RF-EMF and cognitive function in children and adolescents have been inconsistent due to a number of study design-related factors, such as types of exposure and outcome measures, age of participants, and the era of study conduction. The present literature review focused on these possible factors that could explain this inconsistency. This review identified 12 eligible studies (participants ages 4 to 17 years) and extracted a total 477 relations. In total, 86% of the extracted relations were not statistically significant; in the remaining 14%, a negative relation between RF-EMF and cognitive performance was detected under limited conditions: when (1) RF-EMF was assessed using objective measurement not subjective measurement (i.e., questionnaire), (2) participants were relatively older (12 years and above) and had greater opportunity of exposure to RF-EMF, and (3) the collection of cognitive function data was conducted after 2012. Given that 86% of the extracted relations in this analysis were not statistically significant, the interpretation should be approached with caution due to the possibility of the 14% of significant relationships, extracted in this review, representing chance findings.

## 1. Introduction

Over the last two decades, sources of radiofrequency electromagnetic field (RF-EMF) in the high-frequency band, such as mobile phones and wireless local area networks (LANs), have rapidly spread in children’s living environment. The percentage of children and adolescents aged 10–20 years who report owning mobile phones has reached over 70% in the developed countries [1,2], and is still increasing. Even though exposure to RF-EMF is limited in the scope of guidelines (International Commission on Non-Ionizing Radiation Protection, 2020), with the increasing exposure of children and adolescents to RF-EMF, concerns have been raised worldwide about potential adverse health effects of RF-EMF on this age group. Consequently, the World Health Organization (WHO, Geneva, Switzerland) is undertaking a health risk assessment of RF-EMF [3].

As the brain is exposed to RF-EMF while calling with a mobile phone, the initial concern was a possibility of brain tumors [4,5]. However, recently, the adverse effects on neurocognitive function have come up as one of the most important concerns. A previous study demonstrated the possible biological mechanisms behind the influence of RF-EMF on cognitive function [6]. For example, the International Commission on Non-Ionizing Radiation Protection (ICNIRP) 2020 guidelines state that RF-EMFs can affect the body by causing changes in membrane permeability and temperature rise [7].

Given that childhood and adolescence are characterized by extensive changes in brain structure, function, and connectivity [8,9,10,11], clarifying the influence of RF-EMF on cognitive development has been a high priority [3]. A meta-analysis published in 2008 analyzed 10 studies and suggested that electromagnetic fields emitted by Global System for Mobile Communications (GSM) mobile phones (~900 to ~1800 MHz) may have a small, but significant, impact on attention and working memory performance in adult humans [12]. However, the health effects of RF-EMF on cognitive function in children and adolescents are still controversial. Indeed, several previous studies have reported both adverse and favorable relations between RF-EMF and cognitive function in children and adolescents [13,14]. To further clarify the association, we review and summarize the previous epidemiological studies investigating the relation between RF-EMF in the high-frequency band and cognitive function in children and adolescents, and discuss possible factors that could affect the results and cause inconsistencies in findings.

## 2. Materials and Methods

This review conducted the literature search in line with the Preferred Reporting Items for Systematic Reviews and Meta-Analysis (PRISMA) statement [15]. The PRISMA checklist is provided in Table S1.

### 2.1. Literature Search

**Figure 1:**
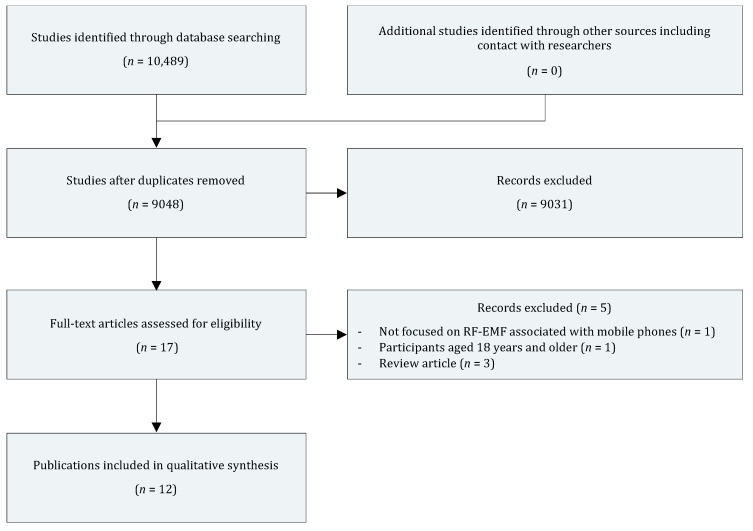
PRISMA (Preferred Reporting Items for Systematic Reviews and Meta-Analysis) flow diagram showing the results of the literature search.

The PRISMA flow diagram showing the results of the literature search is provided in Figure 1. The literature search was performed in December 2019 using the following electronic bibliographic databases: PubMed, Cochrane Library, and Web of Science. The search terms used were as follows: (“adolescents” OR “childhood” OR “children” OR “early childhood” OR “elementary school” OR “fetal” OR “infancy” OR “preadolescent” OR “prenatal” OR “preschool” OR “school” OR “toddler” OR “utero” OR “young adult” OR “youth”) and (“2G” OR “3G” OR “4G” OR “electromagnetic” OR “GSM” OR “Hz” OR “phone” OR “radio waves” OR “radio frequency” OR “radiofrequency” OR “UMTS” OR “wideband”) and (“attention” OR “cognition” OR “cognitive flexibility” OR “cognitive control” OR “cognitive function” OR “cognitive performance” OR “executive function” OR “executive functioning” OR “executive dysfunctions” OR “inhibition” OR “inhibitory control” OR “intelligence” OR “memory” OR “neurocognitive” OR “planning” OR “problem solving” OR “processing speed” OR “shifting” OR “switching” OR “verbal fluency”). Additionally, the reference lists of the relevant studies were also used to locate research articles.

### 2.2. Inclusion and Exclusion Criteria

This review included epidemiological studies that examined the effects of exposure to RF-EMF on cognitive functions in children and adolescents. We included studies that involved exposure to RF-EMF in the context of mobile phones, comprised participants aged 17 years or younger, and were original articles written in English. As we focused on the chronic effects of RF-EMF exposure on cognitive function, studies examining the acute effects of RF-EMF were excluded.

### 2.3. Screening and Data Extraction

The primary author (T.I.) screened the titles and abstracts of the retrieved studies identified by the search strategy; in a second step, these potentially eligible studies were re-evaluated through the full text. The primary author (T.I.) extracted the following data from each candidate article: (i) background (authors, year of publication); (ii) study design; (iii) year of conduction; (iv) characteristics of participants (i.e., sample size, gender, and age); (v) exposure and its assessment method used in the study; (vi) cognitive function assessment used in the study; and (vii) results of outcomes. The data were then checked by the coauthors (K.Y., A.A. and Y.T.).

### 2.4. Quality Assessment

With regard to the study quality, two authors (T.I and K.Y.) independently rated the included studies using the Joanna Briggs Institute critical appraisal checklist [16], and all discrepancies were resolved by consensus. The results of the quality assessment are provided in Table S2.

## 3. Results

### 3.1. Literature Findings

Twelve studies were extracted and included in this review. Table 1 provides a summary of all the studies included in the present analysis.

Eleven studies from five cohorts (Mobile Radiofrequency Phone Exposed Users’ Study [MoRPhEUS], Health Effects Related to Mobile phonE use in adolescentS [HERMES], Examination of Psychological Outcomes in Students using Radiofrequency dEvices [ExPOSURE], Amsterdam Born Children and their Development [ABCD], and INfancia y Medio Ambiente [INMA]) reported the cross-sectional and longitudinal relation between RF-EMF and cognitive function in children and adolescents. One study of three birth-cohorts (Danish National Birth Cohort [DNBC], INMA, and Korean Mothers and Children’s Environment Health [MOCEH]) reported the predictive relation between prenatal RF-EMF and childhood cognitive function.

#### 3.1.1. MoRPhEUS

The MoRPhEUS is an epidemiological study conducted in Australia, examining possible associations between mobile phone exposure and cognitive function in adolescents aged 12–13 years [13,14]. The MoRPhEUS recruited 479 adolescents and was conducted from 2005 to 2007.

Two studies from the MoRPhEUS are the first epidemiological studies that reported the relation between mobile phone exposure and cognitive function in adolescents [13,14]. These studies identified the relation between self-reported exposure to mobile phones (a modified version of the Interphone questionnaire [27]) and cognitive function. The Interphone questionnaire contains seven item, such as “What is the average number of calls you make per week?”, “What is the average number of calls you receive on your mobile phone per week?”, and “What is the average number of text (short message service [SMS]) messages you send and receive per week?” Cognitive function was evaluated using six tasks: signal detection task as a measure of simple reaction time, one card learning task and associative learning task as measures of memory, Stroop task as a measure of interference control, N-back task as a measure of working memory, and moving card monitoring task as a measure of the ability to track and predict the motion of an object.

The cross-sectional study with 317 adolescents was conducted from 2005 to 2006 [13]. Most of the adolescents had used a mobile phone (94%) and owned a personal phone (77%). The total number of voice calls per week was associated with poorer accuracy on the working memory tasks (one-back and two-back tasks) and memory task (associative learning task), shorter reaction time on the memory tasks (one card and associative learning task), and longer completion time on the interference control task (Stroop task), after controlling for age, gender, ethnicity, socioeconomic status, and handedness. There was some evidence that the negative relation between the total number of voice calls per week and accuracy on the working memory tasks. The total number of SMS messages per week was associated with poorer accuracy on the working memory tasks (one-back and two-back tasks) and memory task (associative learning task), and shorter reaction time on the memory task (one card learning task), after controlling for confounders. The authors suggested that mobile phone use is associated with poorer memory, interference control, and working memory, but also with superior reaction time. The authors interpreted that mobile phone usage is associated with faster but less accurate performance on cognitive tasks due to impulsive response style and/or familiarity with computer key pressing.

The longitudinal study with 238 adolescents was conducted in 2005/2006 and a 1-year follow-up was conducted in 2006/2007 [14]. The ownership of mobile phones in adolescents increased from baseline (75%) to follow-up (86%), and the total number of voice calls and SMS per week also increased. The change in the total number of voice calls and SMS per week was remarkable for adolescents with lower numbers of voice calls and SMS at baseline, while for those who already had high numbers of voice calls and SMS at baseline, the numbers at follow-up decreased. The total number of voice calls per week at baseline was associated with a lesser reduction in reaction time on the working memory task (two-back task) and memory task (one card learning task), after controlling for age at baseline, gender, ethnicity, growth, time period between baseline and follow-up, and socioeconomic status. The total number of SMS per week at baseline was associated with a lesser reduction in reaction time on the working memory task (two-back task), after controlling for confounders. An increase in the total number of voice calls per week over the 1-year period was associated with a larger reduction in reaction time on the working memory task (two-back task) and lesser reduction in simple reaction time (signal detection task), after controlling for confounders. Considering the patterns of change in exposure (decrease in adolescents with already high exposure at baseline and vice versa), the authors concluded that the significant negative association in the cross-sectional analysis and positive association in the longitudinal analysis obtained between mobile phone use and cognitive task performance was due to statistical regression to the mean and not due to mobile phone exposure.

#### 3.1.2. HERMES

The HERMES is a prospective cohort study conducted in central Switzerland, to examine whether exposure to RF-EMF affects cognitive functions or causes behavioral problems and non-specific health disturbances in adolescents aged 12–17 years [28]. A total of 895 adolescents (Wave 1: 439 adolescents, participation rate = 36.8%; Wave 2: 456 adolescents, participants rate = not reported) participated in the baseline assessments of the HERMES conducted between June 2012 (Wave 1) and April 2014 (Wave 2); each was followed-up approximately 1 year after baseline [17,18,19].

Three studies from the HERMES study reported a relation between RF-EMF and cognitive function in adolescents [17,18,19]. These studies reported associations between self-reported and objectively recorded RF-EMF and memory and concentration. The duration of data usage on the mobile phone (e.g., for surfing and streaming), duration on own or any other mobile phone, and call duration with cordless (fixed line) phone were collected using a self-reported questionnaire. Objectively recorded mobile phone use data, including the duration of each call and the network (GSM or Universal Mobile Telecommunications System [UMTS]) on which the call started, the number of SMS sent per day, and the volume of data usage (Mega Bite [MB]/day), were obtained from the mobile phone operators. Self-reported data of the duration of gaming on computers and TV and the number of all kinds of text messages (SMS, WhatsApp, etc.) were collected as marginally relevant for RF-EMF. Memory and concentration were evaluated using the Intelligenz-Struktur-Test and Frankfurter Adaptiver Konzentrationsleistungs-Test-II.

The data obtained from Wave 1 [18,19] showed that most of the adolescents owned a mobile phone at baseline (94%), and the percentage slightly increased at follow-up (98%). The adolescents with medium (>50% to <75%) but not high (>75%) operator recorded number of SMS exposure exhibited decreased memory task (figural memory) performance relative to the low exposed adolescents (<median), after controlling for age, gender, nationality, school level, physical activity, alcohol, education of parents, growth, and the time period between baseline and follow-up. The adolescents with median (>50% to <75%) but not high (>75%) RF-EMF dose to the brain, calculated based on self-reported mobile phone call duration, also showed decreased memory task (verbal memory) performance relative to the adolescents with a low dose (<median), after controlling for confounders. Adolescents with high (>75%) RF-EMF dose to the brain and the whole body, calculated based on self-reported mobile phone call duration and objectively recorded mobile phone call duration, showed decreased memory task (figural memory) performance relative to the adolescents with a low dose (<median), after controlling for confounders. The findings demonstrating a negative relation between RF-EMF and memory task performance evaluated by the figural memory task were replicated by the subsequent prospective cohort study using an approximately double sample size by adding the data from Wave 2 [17]. The authors concluded that these findings provided preliminary evidence suggesting that RF-EMF is related to memory performance in adolescents.

Contrary to the above findings, the findings regarding the relation of RF-EMF to concentration capacity were inconsistent [18]. The cross-sectional analyses showed that self-reported and objectively recorded wireless communication devices use and RF-EMF dose were associated with poor concentration capacity, while no such association was found in the longitudinal analyses [18]. Considering the discrepancy between the results from the cross-sectional and longitudinal data analyses, the authors concluded that concentration capacity is not affected by the use of wireless communication devices and RF-EMF exposure.

#### 3.1.3. ExPOSURE

The ExPOSURE is a prospective cohort study conducted in Australia, examining possible relations between mobile phone exposure and cognitive function in children aged 8–11 years [20,21,22]. A total of 619 children participated in baseline assessments of ExPOSURE conducted between November 2010 and February 2012, and were followed-up approximately 1 year after baseline (between March 2012 and March 2013) [20,21,22].

Three studies from the ExPOSURE study reported a relation between mobile phone exposure and cognitive function in children [20,21,22]. These studies examined the relation between self- and parents-reported exposure to mobile phones and cognitive function. Children’s mobile and cordless phone use and the extent of use were reported by their parents, while children reported whether they owned or used a mobile phone. Cognitive function was evaluated using seven tasks: signal detection task as a measure of simple reaction time, identification task as a measure of choice reaction time, one card learning task as a measure of memory, Stroop task as a measure of interference control, go/no-go task as a measure of response inhibition, one-back task as a measure of working memory, and Groton maze learning task as a measure of executive function.

In the cross-sectional analysis conducted with 619 children in mid-2011 [22], parental responses indicated that 31% of the children owned or used a mobile phone at the time of the study, while 80% of the children reported that they used a cordless phone. Mobile phone use was associated with longer reaction time on the response inhibition task (go/no-go task), but this association was found only for boys and not for girls after controlling for age, gender, a language other than English, handedness, and socioeconomic status. Mobile phone use was also associated with slower reaction times on the choice reaction time task (identification task) in boys but not girls. Cordless phone use was associated with longer completion time on the interference control task (Stroop task), and poorer accuracy on the choice reaction time task (identification task) and memory task (one card learning task), after controlling for confounders. The negative association between cordless phone use and interference control task (Stroop task performance) was observed only for girls and not for boys. Considering that only 5 of 78 comparisons were statistically significant, the authors concluded that there was little evidence that cognitive function was consistently associated with cordless phone and mobile phone use in children.

In the prospective cohort analyses with 412 children [20], the percentage of owned or used mobile phone increased from baseline (parental report: 31%; self-report: 57%) to follow-up (parental report: 43%; self-report: 68%). The use of a cordless phone at home was reported for 76% of the children both at baseline and follow-up. The total number of voice calls and SMS per week increased from baseline to follow-up. Increased mobile phone use was associated with larger mean reduction in response time on the response inhibition task (go/no-go task), smaller reduction in the number of total errors on the executive function task (Groton maze learning task), and larger increase in response time on the interference control task (Stroop task), after controlling for age at baseline, gender, ethnicity, SES (classified into quintiles), the time lag between baseline and follow-up, handedness, and total weekly screen time. The increased number of weekly cordless phone calls was associated with a smaller increase in accuracy on the simple reaction time task (detection task) after controlling for confounders. Due to the contradictory results across the cognitive tasks, that is, favorable relation of mobile phone use with response inhibition and adverse relation of mobile phone use with interference control and executive function, the authors concluded that the observed changes in cognitive tasks could be pure chance findings.

#### 3.1.4. ABCD

The ABCD is a prospective birth cohort study conducted in the Netherlands, examining the relation of maternal lifestyle and psychosocial determinants during pregnancy with multiple aspects of development and health of the child [29]. Between January 2003 and March 2004, 12,373 women were approached and 7050 of them filled out the pregnancy questionnaire and granted permission for follow-up.

A single study from the ABCD study reported an association between RF-EMF and cognitive function in children aged 5–6 years [23]. This study showed the relation of mother-reported RF-EMF and residential RF-EMF from mobile phone base stations with cognitive function. When children were 5–6 years old, their cognitive function was evaluated using 3 tasks: baseline speed task as a measure of simple reaction time, response organization task as a measure of inhibitory control and cognitive flexibility, and pursuit and tracking task as a measure of visuomotor coordination. When children were 7 years old, presence or absence of the main residential RF-EMF indoor sources (i.e., cordless phone base stations and Wi-Fi) and the frequency of the child’s cell phone and cordless phone use pertaining to the time point of the cognitive function tests were reported retrospectively by their mothers. Residential RF-EMF exposure from mobile phone base stations was estimated using the 3D geospatial radio wave propagation model NISMap. The downlink component of the three mobile phone communication bands (GSM900, GSM1800, and UMTS) was assessed using a country-wide mobile phone base station data set from 2011.

The cross-sectional study with 2354 children [23] showed that the residential RF-EMF exposure from mobile phone base station was associated with improved reaction time on the inhibitory control and cognitive flexibility task (response organization task), and reduced accuracy on the visuomotor coordination task (tracking task), after controlling for maternal education, area-level indicator of socioeconomic status, country of birth, age, body mass index (BMI), tobacco use, alcohol consumption, depression, anxiety, stress, mother–child attachment, parental financial situation, child’s gender, number of siblings, time playing with computers/video games, and age at cognitive test. Residential presence of RF-EMF indoor source was associated with shorter reaction time and reduced response variability on the simple reaction time task (baseline speed task), improved accuracy on the inhibitory control and cognitive flexibility tasks (response organization task), and improved accuracy and reduced response variability on the visuomotor coordination task (pursuit task), after controlling for confounders. Personal cordless phone use was associated with reduced reaction time on the inhibitory control and cognitive flexibility tasks (response organization task), after controlling for confounders. Personal cell phone use was associated with improved accuracy and reduced response variability on the visuomotor coordination task (pursuit task), after controlling for confounders. From these inconsistent results, that is, both positive and negative relations between RF-EMF and cognitive task performance, the authors concluded that RF-EMF exposure from several sources as well as personal cell phone and cordless phone use did not show a consistent association with cognitive function in children aged 5–6 years.

#### 3.1.5. INMA

The INMA is a prospective birth cohort study conducted in different regions of Spain (Ribera d’Ebre, Menorca, Granada, Valencia, Sabadell, Asturias, and Gipuzkoa), examining the possible role of environmental pollutants in air, water, and diet during pregnancy and early childhood in child growth and development [30]. Recruitment started in March 1997, and 3174 eligible mother–son pairs were recruited [30].

A single study from the INMA study (Granada cohort) demonstrated a relation between objectively measured RF-EMF and cognitive function in boys aged 9–11 years [24]. In the Granada cohort, 668 mother–son pairs were recruited from October 2000 through July 2002 and invited for follow-up assessment when the children reached the age of 9–11 years (2011 to 2012). Spot electric field measurements within the 100 kHz to 6 GHz frequency range were performed in the immediate surroundings of the children’s dwellings using a TS/001/UB Taoma base unit (Tecnoservizi, Rome, Italy) with a TS/004/EHF isotropic electric field probe. Cognitive function was evaluated using the following tasks: Kaufman Brief Intelligence Test (K-BIT) as a measure of general cognitive intelligence based on the composite Intelligence Quotient (IQ), language, and abstract reasoning; Continuous Performance Test (CPT) as a measure of attention; Complutense-Spain Madrid Verbal Learning Test (TAVECI) as a measure of verbal memory; Trail Making Test (TMT) as a measure of visual-motor coordination and cognitive flexibility; two subtests (symbol search and coding) from the Wechsler Intelligence Scale for Children (WISC-IV) as a measure of processing speed; Stroop task as a measure of interference control; go/no-go task as a measure of response inhibition; letter–number sequencing subtest from the WISC-IV as a measure of working memory; and categorical verbal fluency test as a measure of verbal fluency.

The cross-sectional study with 123 boys [24] showed that the boys living in higher RF-EMF areas had lower IQ and language (K-BIT) scores after controlling for children’s place of residence, mother’s smoking during pregnancy, maternal schooling, and Wi-Fi. No other association was detected. Although RF-EMF has a negative relation with IQ and language in children, the authors submitted that definitive conclusions could not be drawn, as the majority of the performance on the cognitive tasks was not associated with RF-EMF.

#### 3.1.6. Combined Results from DNBC, INMA, and MOCEH

A single study examined the relation of subjectively measured RF-EMF during pregnancy with cognitive function in children aged 4–6 years [25]. The study analyzed combined data from three cohort studies, namely, DNBC (Denmark), INMA (Spain), and MOCEH (Korea). Cell phone use among mothers was assessed during pregnancy (INMA and MOCEH) or recalled by mothers when the children reached 7 years of age (DNBC). The Wechsler Preschool and Primary Scale of Intelligence–Revised (WPPSI–R; DNBC and MOCEH) and McCarthy Scales of Children’s Abilities (MSCA; INMA) were used to assess children’s general cognition, verbal cognition, and non-verbal cognition.

The results of the three birth cohort studies with a combined total of 3089 children [25] showed no statistically significant associations between the frequency of prenatal cell phone use and children’s cognitive function scores, after controlling for gender of the child, age of the child, maternal IQ, maternal age, parity, mother’s history of psychological distress, maternal education, paternal education, prenatal smoking, prenatal alcohol use, and maternal pre-pregnancy BMI. However, there was a general pattern of lower scores on verbal, non-verbal, and overall cognition with a higher frequency of prenatal cell phone use. The authors concluded that although they observed patterns of lower mean cognition scores among children in relation to high frequency maternal prenatal cell phone use, the causal nature and mechanism of this relation remain unknown because maternal cell phone use could be a proxy for other factors related to child cognition (e.g., parenting behaviors, socioeconomic status, and the child’s own technology use behaviors).

#### 3.1.7. Cross-Sectional Study Using Objective Exposure Measurement

A single cross-sectional study reported the relation of objectively measured RF-EMF in the high-frequency band with cognitive function in adolescents [26]. The cross-sectional study with 217 adolescents aged 13–16 years [26] reported a relation between RF-EMF exposure at school monitored by the Narda Safety Test Solution SRM-3006 and cognitive function measured by 2 tasks: Motor Screening task as a measure of simple reaction time and Spatial Working Memory task as a measure of working memory. Children who were exposed to high RF-EMF produced by mobile base stations had poorer performance on the simple reaction time and working memory tasks (Motor Screening task and Spatial Working Memory task). The authors concluded that objectively measured RF-EMF was associated with poor cognitive function in children and adolescents.

#### 3.1.8. Summary

Overall, the results from previous studies regarding the relation between RF-EMF in the high-frequency band and cognitive function in children and adolescents have been inconsistent. Previous studies have reported both negative and positive relations between RF-EMF in the high-frequency band and cognitive task performance. Most of the associations analyzed were not statistically significant. These contradictory results might be due to confounding factors (impulsive response style and familiarity to computer key pressing), awareness and/or recall bias of self-reported RF-EMF, or statistical phenomena (such as statistical regression to the mean and pure chance findings). Hence, there is still no evidence regarding whether or not RF-EMF in the high-frequency band affects cognitive function in children and adolescents.

### 3.2. Possible Explanations for the Contradictory Results

To suggest future research directions to resolve the contradictory findings showing both negative and positive relations between RF-EMF in the high-frequency band and cognitive task performance in children and adolescents, it is useful to discuss how the various study designs could affect the results. To this end, we extracted all associations reported by the above 12 studies and explored the differences among the results in each study condition. The primary author (T.I.) screened and extracted a total of 477 relations between RF-EMF in the high-frequency band and cognitive task performance gathered from the abovementioned 12 studies. The data were then checked by the coauthors (K.Y., A.A. and Y.T.). Sixty-seven (14%) of the extracted relations were significant; of these, 51 (76%) relations were negative and 16 (24%) were positive. To unify the criteria of significance across the studies and objectively conduct the review, we focused only on these significant associations. The details of all the relations included in this review are provided as Data S1 with this paper.

#### 3.2.1. Familiarity and Training Effects of Computer Usage

In the WHO research agenda, to distinguish the “training” of motor and neuropsychological skills caused by the use of a mobile phone from the effects of RF-EMF is considered as one of the challenges for neuropsychological studies [3]. Previous studies reported that certain game play (action, shooting, and driving) improves processing speed [31,32,33]. Accordingly, familiarity and training effects of computer key pressing might cancel the negative relation, or cause the positive relation, of RF-EMF to cognitive task performance. However, only 1 of the 12 studies included familiarity with computer games as a confounding variable [23]. A previous study reported that the greater familiarity with computer games was significantly associated with higher performance on the visual retention test and pursuit aiming test [34]. Given that video gaming improves reaction time without reductions in accuracy [32], the confounding effects of familiarity and the training effects of computer games on cognitive task performance may disproportionately be observed for reaction time relative to accuracy or other scoring methods.

**Figure 2:**
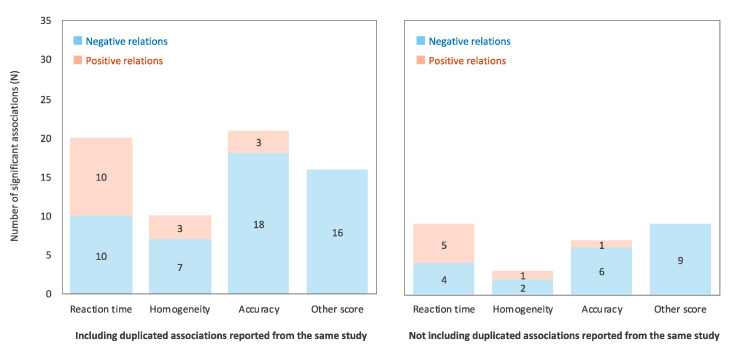
The proportion of negative and positive relations among the significant relations between RF-EMF (radiofrequency electromagnetic field) and cognitive task performance in each index. Cases including duplicated associations reported from the same study (**left** panel). Cases not including duplicated associations reported from the same study (**right** panel). The negative relation indicates the relations of RF-EMF exposure to poor cognitive task performance (i.e., longer reaction time, lower homogeneity, and poor accuracy and other scores), while positive relation indicates the opposite.

The outcome measures were divided into four categories: reaction time, homogeneity, accuracy, and other scores. Homogeneity refers to intra-individual response variability (i.e., a within-subject standard deviation of the reaction time), which is known as a marker of cognitive and brain health [35]. Other scores included scores on standardized tests (such as the K-BIT and Intelligenz-Struktur-Test); interference score on Stroop task; and Neurobehavioral Ability Index calculated from reaction time, homogeneity, and accuracy. Then, the proportions of negative and positive relations among the significant relations in each index were aggregated (Figure 2). Note that a negative relation indicates the relation between RF-EMF exposure and poor cognitive task performance (i.e., longer reaction time, lower homogeneity, and poor accuracy and other scores), while a positive relation indicates the opposite. The results showed that the relation between RF-EMF and reaction time was inconsistent relative to homogeneity, accuracy, and other scores. These patterns were consistent in cases not including duplicated associations, reported from the same study (Figure 2). This means the current results were not biased by double-counting multiple associations from the same study.

Considering that video gaming improves reaction time without decreasing accuracy [32], the relation between RF-EMF and cognitive task performance in children and adolescents may be inconsistent in terms of the reaction time on the cognitive task relative to the other indices. Thus, when significant relations between RF-EMF measures and reaction time on the cognitive tasks are detected in future research, the results should be interpreted with caution. To address the issue of training effects, future studies need to manipulate cognitive tasks. For example, studies could implement sufficient practice versions of the cognitive task to reduce the confounding influence of learning effects. Furthermore, cognitive tasks can be calibrated to each individual’s level of cognitive performance to improve the sensitivity of the task [36].

#### 3.2.2. Self-Reported Radiofrequency Wave Exposure

As many previous studies have pointed out [13,14,20,21,22,25], self-reported evaluation of exposure is a limitation in the research on RF-EMF–cognitive function interaction in children and adolescents. The self-reported RF-EMF includes the self-reported and/or parents-reported usage of cell phone, cordless phone, mobile phone, and media devices related to RF-EMF. Self-reported RF-EMF assessment causes awareness bias [37,38]. A previous study reported that participants recalled their phone use with moderate systematic error and substantial random error [38]. The validity of the self-reported number of calls per day was adequate, while that of the average duration of each call was moderate [37]. Although call duration is important for estimating RF-EMF in children and adolescents independent of the number of voice calls, several studies excluded call duration measures as an exposure measure due to low validity [14,21].

In addition, self-reported RF-EMF through questionnaires does not allow for the relation between RF-EMF and cognitive function to be examined, independent of the various confounding factors. For example, RF-EMF measured subjectively via questions regarding mobile phone and other media ownership and usage is associated with screen time and computer gaming. As mentioned above, certain game play improves the processing speed [32]. Accordingly, the use of self-reported measures could reduce the power to detect a negative relation between cognitive task performance and RF-EMF due to mobile phone use in children and adolescents.

**Figure 3:**
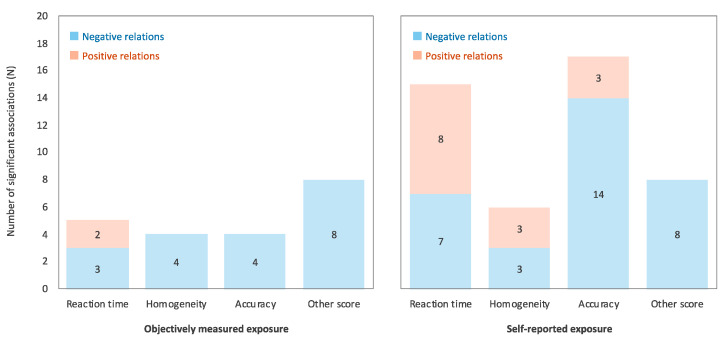
The proportions of negative and positive relations among the significant relations between RF-EMF and cognitive task performance in objectively measured (**left** panel) and self-reported exposure (**right** panel). The negative relation indicates the relations of RF-EMF exposure to poor cognitive task performance (i.e., longer reaction time, lower homogeneity, and poor accuracy and other scores), while positive relation indicates the opposite.

The proportions of negative and positive relations among the significant relations in objectively measured and self-reported exposure were aggregated separately (Figure 3). The results showed that the relation between self-reported RF-EMF and cognitive function was inconsistent relative to that between objectively measured RF-EMF and cognitive function. In addition, all associations except reaction time were negative when RF-EMF was objectively measured. Considering that self-reported RF-EMF assessment causes awareness bias [37,38], and is inseparable from familiarity and training effects of computer usage, the relation between RF-EMF and cognitive function in children and adolescents may be inconsistent when exposure measures are evaluated with the self-reported questionnaires. Thus, objective exposure measurements, such as RF-EMF from mobile phone base station and indoor source, and media usage related to RF-EMF obtained from operator data, should be used in future studies, whenever possible.

#### 3.2.3. Participants’ Age

The effects of RF-EMF on cognitive function in children and adolescents could be moderated by age. The range of the participants’ age across the studies included in this review was large (i.e., 4 to 17 years). The MoRPhEUS study reported that most of the adolescents aged 12–13 years had used a mobile phone (94%) and owned a personal phone (77%) [13]. Similarly, the HERMES study reported that most of the adolescents aged 12–17 years owned a mobile phone at baseline (94%), and the percentage slightly increased at follow-up (98%) [19]. Relative to these numbers, the percentage of owning or using a mobile phone in younger children aged 8–11 years was quite low (31% according to parents report and 57% as per self-report) [20]. Given the high likelihood of exposure to RF-EMF from mobile phone in adolescents relative to children, the effects of RF-EMF on cognitive function could be moderated by the participants’ age. From the perspective of brain development, the effects of RF-EMF may be moderated by the participants’ age due to different periods of development characterized by extensive changes in brain structure, function, and connectivity [8,9,10,11] and its region- and function-specific developmental trajectories [39,40]. In addition, variations in cognitive performance may also be moderated by differences in development stage.

**Figure 4:**
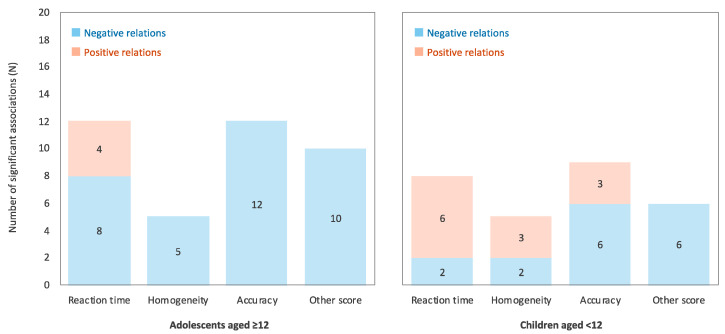
The proportions of negative and positive relations among the significant relations between RF-EMF and cognitive task performance in adolescents (aged ≥12 years: **left** panel) and children (aged <12 years: **right** panel). The negative relation indicates the relations of RF-EMF exposure to poor cognitive task performance (i.e., longer reaction time, lower homogeneity, and poor accuracy and other scores), while positive relation indicates the opposite.

The proportions of negative and positive relations among the significant relations in adolescents (aged ≥12 years) and children (aged <12 years) were aggregated separately (Figure 4). The results showed that the relation between RF-EMF and cognitive task performance was inconsistent in children relative to adolescents. In addition, all associations except reaction time were negative in adolescents aged 12 years and above. Given that the opportunities of being exposed to RF-EMF are more frequent in adolescents than in children [13,19,20], the relationship between RF-EMF and cognitive function could be disproportionately greater in adolescents, if there is a threshold on the effects of RF-EMF on cognitive function. Another possible explanation is the different periods of development characterized by extensive changes in brain structure, function, and connectivity [8,9,10,11]. Thus, the participants’ age may be one of the moderators in the influence of RF-EMF on cognitive function.

#### 3.2.4. Era of Study Conduction

The strength of the effects of RF-EMF exposure on cognitive function in children and adolescents could have changed with the era. Technological advances have dramatically increased the sources of RF-EMF in children’s living environment but lowered the level of exposure to RF-EMF from each device. For example, fourth-generation communication long-term evolution (4G-LTE) was deployed in Norway and Stockholm in 2009 and in the United States by Verizon in 2011, and was subsequently rapidly introduced throughout the world. The 4G-LTE system has provided a very fast Internet speed over the current radiofrequency rate, and increased the chances of exposure to RF-EMF via new technologies such as high-quality video streaming. On the other hand, exposure to RF-EMF from devices has reduced with technological advances. For example, compared to 2G, the introduction of 3G has lowered RF-EMF exposure of mobile phone users. Therefore, the increased sources of RF-EMF could have strengthened the effects of RF-EMF exposure on cognitive function. On the contrary, from the perspective of lowered exposure to RF-EMF from each device, the effects of exposure to RF-EMF on cognitive function could have weakened. Together, although, we could not set a clear hypothesis regarding the direction of the change, the effects of RF-EMF on cognitive function could have changed with the era.

**Figure 5:**
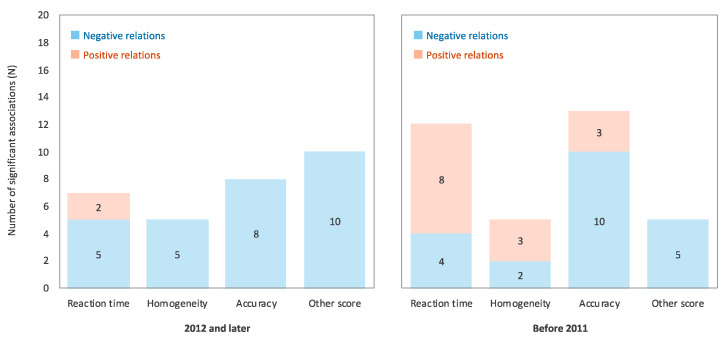
The proportions of negative and positive relations among the significant relations between RF-EMF and cognitive task performance during 2012 and later (**left** panel) and before 2011 (**right** panel); 2 relations from Meo et al. [26] were excluded because the year of conduction was not reported. The negative relation indicates the relations of RF-EMF exposure to poor cognitive task performance (i.e., longer reaction time, lower homogeneity, and poor accuracy and other scores), while positive relation indicates the opposite.

For this analysis, the extracted relations were divided into two eras based on the years of cognitive assessment (before 2011, and 2012 and later, i.e., the median years of extracted data), and the proportions of negative and positive relations among the significant relations in each era were aggregated separately (Figure 5). The results showed that the relation between RF-EMF and cognitive task performance was inconsistent before 2011 relative to 2012 and later. In addition, all relations except reaction time were negative in 2012 and later years. While the exposure to RF-EMF from each device has lowered with technological advances, as the opportunity of being exposed to RF-EMF and its sources has changed in the last decade, the effects of RF-EMF on cognitive function could be disproportionately greater in recent years relative to before 2011. Thus, monitoring the secular trend of the influence of RF-EMF on cognitive function is needed.

## 4. Conclusions

Over the past decade, several studies have attempted to clarify the effects of RF-EMF in the high-frequency band on cognitive function in children and adolescents. However, there is still no consensus regarding whether RF-EMF in the high-frequency band affects cognitive function in children and adolescents as evidenced by contradictory findings showing both negative and positive relations between RF-EMF in the high-frequency band and cognitive task performance. This review offers some possible explanations for the contradictory results. The results from data aggregation suggest that the reaction time may be strongly confounded by familiarity and training effects of computer usage and could lead to contradictory results. The results of the negative relation between RF-EMF in the high-frequency band and cognitive task performance have been detected consistently under specific conditions: (1) when RF-EMF was assessed using objective measurements, (2) when the participants were relatively older (aged ≥12 years) with greater opportunity of exposure to RF-EMF, and (3) when the cognitive function data were collected in 2012 or later. The possible explanations for these findings are the awareness bias of the self-reported RF-EMF, age differences leading to differences in the amount of opportunity of exposure to RF-EMF, and the dramatic changes in the opportunity of being exposed to RF-EMF and its sources. However, note that we focused only on the statistically significant associations and these associations were merely 14% of all extracted associations. Given that the remaining 86% of the extracted relations were not statistically significant, the interpretation should be approached with caution due to the possibility of the 14% of significant relationships, extracted in this review, representing chance findings. This means exposure to RF-EMF may not influence cognitive performance in children and adolescents. This review identifies and discusses the conditions that could detect the consistent direction of the relations when the associations are statistically significant. In the future, a meta-analytic review analyzing all associations including non-significant relations is needed.

## Figures and Tables

**Table 1:** Summary of all studies included in this review.

First Author	PY	Study Design	Conducted Year	Participants	Exposure	Cognitive Task	Outcome	Main Findings
Abramson et al. [13]	2009	Cross-sectional	Data were collected during 2005 and 2006.	317 students 11–14 years old From MoRPhEUS	Mobile phone usage was assessed using questionnaire completed by children and their parents.	Signal detection task Moving card monitoring task One card learning task Associative learning task Stroop task N-back task	Simple reaction Choice reaction Attention Visual memory Associative memory Interference control Working memory	Mobile phone use was associated with poor accuracy on the N-back task, shorter reaction time on the simple reaction time task, poor accuracy and shorter reaction time on the associative learning task, and longer completion time on the Stroop task.
Thomas et al. [14]	2010	Prospective cohort	Baseline data were collected during 2005 and 2006. Follow-up investigations were conducted approximately 1 year after baseline.	236 students 12–13 years old From MoRPhEUS	Mobile phone usage was assessed using self-reported questionnaire.	Signal detection task Moving card monitoring task One card learning task Associative learning task Stroop task N-back task	Simple reaction Choice reaction Attention Visual memory Associative memory Interference control Working memory	Mobile phone use at baseline was associated with smaller reductions in response times on the 2-back task and One card learning task. Increased mobile phone use was associated with smaller reductions in reaction time on the simple reaction task and larger reductions in reaction time on the 2-back task.
Foerster et al. [17]	2018	Prospective cohort	Baseline data were collected in June 2012 (1st wave) and April 2014 (2nd wave). Follow-up investigations were conducted approximately 1 year after each baseline until April 2016.	669–676 children 10–17 years old From HERMES study	Daily quantitative mobile phone use was obtained from the mobile phone network operators. Mobile phone and other wireless communication devices use was assessed using self-reported questionnaire.	Intelligenz-Struktur-Test	Figural memory Verbal memory	Increase in estimated cumulative RF-EMF brain dose scores was associated with decreased figural memory score.
Roser et al. [18]	2016	Prospective cohort	Baseline data were collected between June 2012 and February 2013. Follow-up investigations were conducted approximately 1 year after baseline.	439 adolescents 12–17 years old From HERMES study	Daily quantitative mobile phone use was obtained from the mobile phone network operators. Mobile phone and other wireless communication devices use was assessed using self-reported questionnaire.	FAKT-II	Concentration	In the cross-sectional analysis, mobile phone usage and cumulative RF-EMF dose were associated with poor FAKT- II performance, while no such association was found in the longitudinal analysis.
Schoeni et al. [19]	2015	Prospective cohort	Baseline data were collected between June 2012 and February 2013. Follow-up investigations were conducted approximately 1 year after baseline.	234 adolescents 7th, 8th, and 9th grades From HERMES study	Daily quantitative mobile phone use was obtained from the mobile phone network operators. Mobile phone and other wireless communication devices use was assessed using self-reported questionnaire.	Intelligenz-Struktur-Test	Figural memory Verbal memory	An increase in mobile phone call duration was associated with a decrease in figural memory. Cumulative RF-EMF brain and whole-body dose were associated with decreases in figural memory scores.
Bhatt et al. [20]	2017	Prospective cohort	Data were collected in November 2010–February 2012 (baseline) and March 2012–March 2013 (follow-up).	412 children 4th grade (9 or 10 years old) From ExPOSURE study	Mobile phone and cordless phone voice calls and number of text message or SMS were assessed using self-reported questionnaire.	Signal detection task Identification task One card learning task Groton maze learning task Go/No-go task Stroop task One-back task	Simple reaction Choice reaction Memory Executive function Response inhibition Interference control Working memory	Increase in mobile phone usage was associated with larger reduction in reaction time in the Go/No-go task, smaller reduction in the errors on the Groton maze learning task, and larger increase in reaction time on the Stroop task. Increase in cordless phone usage was associated with smaller reduction in accuracy on the Signal detection task.
Brzozek et al. [21]	2019	Prospective cohort	Data were collected twice, approximately one year apart between 2011 and 2013.	412 children 4th grade (9 or 10 years old) From ExPOSURE study	Mobile phone use was assessed using self-reported questionnaire.	Signal detection task Identification task One card learning task Groton maze learning task Go/No-go task Stroop task One-back task	Simple reaction Choice reaction Memory Executive function Response inhibition Interference control Working memory	Mobile phone calls were associated with shorter reaction time in the Go/No-go task, lower accuracy on the Groton maze learning task, and poor Stroop task performance.
Redmayne et al. [22]	2016	Cross-sectional	Data were collected in mid-2011.	575–589 children 8–11 years old From ExPOSURE study	Mobile phone and cordless phone use were assessed via the parents’ answers to the questionnaires. The ownership of mobile phone was assessed using self-reported questionnaire.	One-back task Signal detection task Identification task One card learning task Groton maze learning task Go/No-go task Stroop task	Simple reaction Choice reaction Memory Executive function Response inhibition Interference control Working memory	Mobile phone usage was associated with slower reaction time on the Go/No-go task. Cordless phone usage was associated with slower reaction time on the Stroop task, and lower accuracy on the One card learning task and Identification task.
Guxens et al. [23]	2016	Cross-sectional	Between January 2003 and March 2004, participants’ mothers were enrolled during their first prenatal visit to an obstetric care provider.	2354 children 5–6 years old From ABCD study	RF-EMF (mobile phone base station) was estimated using the 3D geospatial radio wave propagation model NISMap. RF-EMF (indoor source, i.e., cordless phone base stations and Wi-Fi) was assessed using questionnaires answered by the mothers. Cell phone and cordless phone use was assessed using questionnaires answered by the mothers (Assessed when children were 7 years old).	Baseline speed task Response organization task Pursuit task Tracking task (Assessed at 5–6 years)	Simple reaction Inhibitory control Cognitive flexibility Visuomotor coordination	Residential RF-EMF exposure from mobile phone base station was associated with improved inhibitory control and cognitive flexibility and reduced visuomotor coordination. Residential presence of RF-EMF indoor source was associated with improved simple reaction time, inhibitory control, and visuomotor coordination. Personal cordless phone use was associated with reduced inhibitory control and cognitive flexibility and improved visuomotor coordination.
Calvente et al. [24]	2016	Cross-sectional	Participants were recruited at birth from 2000 through 2002, were evaluated at the age of 9–11 years.	123 boys 9–11 years old From INMA-Granada cohort	Spot electric field measurements within the 100 kHz to 6 GHz frequency range were performed in the immediate surroundings of children’s dwellings.	K-BIT Letter-number sequencing Categorical verbal fluency Continuous Performance test TAVECI Trail Making Test A WISC-IV Go/No-go task Stroop task Trail Making Test B	IQ Language Executive function Working memory Verbal fluency Attention Memory Visuomotor coordination Simple reaction Response inhibition Interference control Cognitive flexibility	Children living in higher RF exposure areas had lower IQ and language scores.
Sudan et al. [25]	2018	Prospective birth cohorts	Time periods of enrollment in each cohort were: 1996–2002 (DNBC), 2003–2008 (INMA), and 2006–2011 (MOCEH). Cognitive assessment was performed at the age of 60–64 months (DNBC), 49–82 months (INMA), and 47–77 months (MOCEH).	3089 children 4–6 years old From DNBC, INMA, and MOCEH study	Cell phone use among mothers was assessed during pregnancy (Spain and Korea). Cell phone use during pregnancy was recalled by mothers when the children reached 7 years of age (Denmark).	WPPSI-R/MSCA	General cognition Verbal cognition Non-verbal cognition	No associations were found between prenatal cell phone use and WPPSI-R/MSCA scores.
Meo et al. [26]	2018	Cross-sectional	NA	217 students 13–16 years old	RF-EMF exposure at school was monitored by the Narda Safety Test Solution SRM-3006.	Motor Screening task Spatial Working Memory task	Simple reaction Working memory	Children who were exposed to high RF-EMF produced by mobile base stations had poorer performance on the Motor Screening task and Spatial Working Memory task.

Note: FAKT-II = Frankfurter Adaptiver Konzentrationsleistungs-Test II; IQ = intelligence quotient; K-BIT = Kaufman Brief Intelligence Test; PY = publication year; RF-EMF = radiofrequency electromagnetic field; SMS = short message service; TAVECI = Test de Aprendizaje Verbal España-Complutense infantil; WISC-IV = Wechsler Intelligence Scale for Children 4th edition; WPPSI-R = Wechsler Preschool and Primary Scale of Intelligence-Revised; MSCA = McCarthy Scales of Children’s Abilities.

## Supplementary Materials

The following are available online at https://www.mdpi.com/1660-4601/17/24/9179/s1, Table S1: PRISMA checklist, Table S2: Results of quality assessments, Data S1: relations included in this review.
