# Supplementary material for: Exposure to Radiofrequency Electromagnetic Field in the High-Frequency Band and Cognitive Function in Children and Adolescents: A Literature Review
Source: Int J Environ Res Public Health. 2020 Dec 8;17(24):9179. doi: 10.3390/ijerph17249179 (PMC7764655; doi:10.3390/ijerph17249179)
Supplement: Supplementary file 1 [file ijerph-17-09179-s001.zip › Supplementary_Files/Table_S2.docx]

Table S2. Results of the quality assessment

| **First author** | **Q1** | **Q2** | **Q3** | **Q4** | **Q5** | **Q6** | **Q7** | **Q8** |
| --- | --- | --- | --- | --- | --- | --- | --- | --- |
| Abramson et al. [13] | Yes | Yes | Yes | NA | Yes | Yes | Yes | Yes |
| Thomas et al. [14] | Yes | Yes | Yes | NA | Yes | Yes | Yes | Yes |
| Foerster et al. [17] | Yes | Yes | Yes | Yes | Yes | Yes | Yes | Yes |
| Roser et al. [18] | No | Yes | Yes | NA | Yes | Yes | Yes | Yes |
| Schoeni et al. [19] | Yes | Yes | Yes | Yes | Yes | Yes | Yes | Yes |
| Bhatt et al. [20] | Yes | Yes | Yes | Yes | Yes | Yes | Yes | Yes |
| Brzozek et al. [21] | Yes | Yes | Yes | Yes | Yes | Yes | Yes | Yes |
| Redmayne et al. [22] | Yes | Yes | Yes | Yes | Yes | Yes | Yes | Yes |
| Guxens et al. [23] | Yes | Yes | Yes | Yes | Yes | Yes | Yes | Yes |
| Calvente et al. [24] | Yes | Yes | Yes | Yes | Yes | Yes | Yes | Yes |
| Sudan et al. [25] | Yes | Yes | NA | Yes | Yes | Yes | NA | Yes |
| Meo et al. [26] | Yes | Yes | Yes | Yes | No | Yes | Yes | Yes |

Q1. Were the criteria for inclusion in the sample clearly defined?; Q2. Were the study subjects and the setting described in detail?; Q3. Was the exposure measured in a valid and reliable way?; Q4. Were objective, standard criteria used for measurement of the condition?; Q5. Were confounding factors identified?; Q6. Were strategies to deal with confounding factors stated?; Q7. Were the outcomes measured in a valid and reliable way?; Q8. Was appropriate statistical analysis used?
